# Small Anellovirus Infections in Korean Children

**DOI:** 10.3201/eid1305.061149

**Published:** 2007-05

**Authors:** Ju-Young Chung, Tae Hee Han, Ja Wook Koo, Sang Woo Kim, Jeong Kee Seo, Eung Soo Hwang

**Affiliations:** * Inje University, Seoul, Republic of Korea; † Seoul National College of Medicine, Seoul, Republic of Korea

**Keywords:** anellovirus, Republic of Korea, children, letter

**To the Editor:** Recently, Jones et al. (*1*) identified circular DNA sequences, classified as *Anellovirus* genus, in plasma from patients with acute viral infection syndromes. These anelloviruses were then labeled as “small anellovirus (SAV)” because of their smaller genomes when compared with Torque Teno Virus (TTV) and Torque Teno Mini Virus (TTMV), which have small, circular, single-stranded DNA genomes. Although anelloviruses are not associated with any specific disease, TTV has been suggested to play a role in acute respiratory disease (ARD) and in asthma of children (*2*,*3*).

Kawasaki disease and Henoch-Schonlein purpura are important vasculitis disorders in children, possibly triggered by unknown infectious agents. Recently, Gergely et al. (*4*) reported that molecular mimicry involving TTV and the generation of autoantibodies may have a role in the pathogenesis of systemic lupus erythematosus. The purpose of our study was to investigate the prevalence of SAV and its association with various clinical diseases in children.

The study population comprised 81 serum samples from healthy children and 151 serum samples from children hospitalized with hepatitis (81 cases), ARD (40 cases), Kawasaki disease (12 cases), or Henoch-Schonlein purpura (18 cases) during the period January 2002–June 2006. Nasopharyngeal aspirates paired with serum samples were collected from 34 children with ARD, including upper respiratory tract infections, pneumonia, and acute bronchiolitis. Samples were collected after informed consent was obtained at admission from patients’ parents.

PCRs for SAV were performed to amplify a 5′ noncoding region of SAV with specific primers, as described previously (*5*). PCR products were directly sequenced, and nucleotide sequences were registered in GenBank (accession nos. DQ978791–DQ9788810). The χ2 test with Yates correction and Mann-Whitney U-test were used for statistical comparison by using MedCalc (MedCalc Software, Mariakerke, Belgium). A p value <0.05 was defined as statistically significant.

In our study population, serum SAV DNA was detected in 28 (34.5%) of 81 children in the control group and in 66 (43.7%) of 151 children in the disease group. In the healthy control group, the SAV-positive rate was 7.4% (6/81) in children <12 months of age, 16.0% (13/81) in children 1–4 years of age, and 11.1% (9/81) in children 5–15 years of age. In the disease group, the SAV-positive rate was 35.8% (29/81) in patients with hepatitis, 67.5% (27/40) in ARD, 50% (6/12) in Kawasaki disease, and 22.2% (4/18) in Henoch-Schonlein purpura, respectively (Table). Among 34 nasopharyngeal aspirates collected from children with ARD, SAV DNA was detected in 19 (55.9%). Codetection of SAV and respiratory syncytial virus in nasopharyngeal aspirates was observed in 4 patients.

**Table:** Prevalence of SAV viremia in the study population, Republic of Korea, January 2002–June 2006*

Group	Sex (M/F)	Age range (mean age)	No. tested	No. positive (%)	p value
Control	48/33	1 mo–15 y (47 mo)	81	28 (34.5)	
Hepatitis	44/37	1 mo–15 y (58 mo)	81	29 (35.8)	1
HBV	8/12	1–15 y	20	10 (50)	0.30
HCV	11/0	3–10 y	11	5 (45.4)	0.71
Others	10/9	0–13 y	19	7 (36.8)	0.93
Unknown	15/16	0–13 y	31	7 (22.5)	0.3
ARD	22/18	0–5 y (18 mo)	40	27 (67.5)	0.001†
Vasculitis	22/8	1–10 y (46 mo)	30	10 (33.3)	0.91
KD	9/3	1–9 y	12	6 (50)	0.47
HSP	13/5	2–10 y	18	4 (22.2)	0.46
Total	136/96	1 mo–15 y	232	94 (40.5)	

*SAV, small anellovirus; HBV, hepatitis B virus; HCV, hepatitis C virus; ARD, acute respiratory tract disease; KD, Kawasaki disease; HSP, Henoch-Schonlein purpura. †p<0.05, statistically significant.

Percent similarity of nucleotide sequence of PCR products was 99% among SAV isolates. To our knowledge, this is the first report of SAV infections in children. The prevalence and role of SAV in clinical diseases have yet to be determined. Recently, Biagini et al. (*5*) reported that the prevalence of SAV infection was 20% (12/60) in French blood donors. In an Italian study (*6*), the positive rate of SAV DNA was 9.1% (5/55) in patients with hepatitis C compared with 8.6% (3/35) in healthy controls. Thus, the prevalence of SAV in Korean children is much higher than rates reported in adults from other countries (*5*,*6*). Further studies are needed to confirm this finding.

In our study, the prevalence of SAV did not differ significantly between the hepatitis group and the healthy control group. Our results indicate that SAV presence does not appear to have a defining role in hepatitis, as do TTV or TTMV infection (*7*). In a previous study, several groups of viruses including TTV were ruled out as etiologic agents of Kawasaki disease (*8*), findings similar to those of our study. We found that the prevalence of SAV was significantly higher in ARD and that SAV-positive serum results were consistent with those of nasopharyngeal aspirates in 76% (26/34). These findings suggest that the respiratory tract may be a transmission route of SAV in children.

In conclusion, we confirmed the presence of SAV in serum samples and nasopharyngeal aspirates from Korean children. A significantly higher detection of SAV DNA was observed in children with ARD compared with healthy children or children with other clinical diseases.
